# A general mechanism of airborne hearing in recent and early non-tympanate tetrapods

**DOI:** 10.1242/jeb.251719

**Published:** 2026-06-03

**Authors:** Jakob Christensen-Dalsgaard, Tanya Bojesen Lauridsen, Grace Capshaw, Catherine E. Carr

**Affiliations:** ^1^ Department of Biology, University of Southern Denmark, Campusvej 55, DK-5230 Odense M, Denmark; ^2^ Terrariet Reptile Zoo, Kirkehelle 4, DK-5793 Vissenbjerg, Denmark; ^3^ Department of Biology, University of Maryland, College Park, MD 20742, USA; ^4^ Department of Psychological and Brain Sciences, Johns Hopkins University, Baltimore, MD 21218, USA

**Keywords:** Non-tympanic hearing, Tetrapod ear evolution, Earless frogs, Snakes, Biophysics, Salamanders

## Abstract

Tetrapod tympanic hearing probably emerged in the Triassic with independent origins of middle ear structures in each of the major groups, more than 120 Myr after the origin of tetrapods. During this period, any auditory sensitivity must have been based on non-tympanic mechanisms. We focused on the simplest model for non-tympanic hearing: that sound translates the head, and that this vibration is transduced by the inner ear. This is the mode of human low-frequency bone conduction sensitivity and is also the mode of underwater auditory stimulation for most fishes. The efficiency of translation of an object by sound depends on its density and *ka*, the product of the acoustic wavenumber (*k*) and the radius (*a*) of the head. Analytic and simple finite-element models of translation show that head vibration velocities largely are determined by *ka* and density (for objects of the same shape and composition), and are almost constant (between 4 and 5 μm s^−1^ Pa^−1^; neglecting friction) for objects with *ka*<1. We compared sensitivity to sound and to head vibrations in animals lacking tympanic middle ears (snakes, salamanders, earless frogs and lungfish) and showed that the low-frequency airborne sound sensitivity in these species is largely consistent with a translation mechanism. Stimulation of the inner ear by sound translation is likely by an inertial system like the otolithic/otoconial ears of fish and early tetrapods, or by fluid inertia in the inner ear generating hydrodynamic waves that stimulate the hair cells, providing a simple mode of sound reception in earless animals.

## Teaser

**Summary:** We present a simple model for airborne sound reception in tetrapods without specialized accessory structures such as middle ears based on whole-body movement of the animal by sound.

## INTRODUCTION

The tympanic ear is traditionally described as an impedance matching device that counteracts the reflection of sound, otherwise caused by the large impedance difference between the air medium and animal tissue (roughly with the same impedance as water). More accurately, middle ear function is impedance transformation, because sound energy is collected at the tympanum and transformed into another form of mechanical energy (Christensen-Dalsgaard and Manley, 2013) that, in a sensitive middle ear, will generate high-amplitude vibrations at the oval window of the inner ear. The effects of this transformation are large; in humans with a non-functional middle ear, conductive hearing loss results in a decrease in sensitivity by up to 50 dB (Feldman, 1963). Thus, the tympanic middle ear of tetrapods is often seen as a prerequisite for sensitive hearing in air.

However, tympanic middle ears emerged late in tetrapod evolution – in the Triassic, approximately 120 Myr after the origin of tetrapods – and were acquired independently in all the major groups (Clack, 1997; Christensen-Dalsgaard and Carr, 2008; Capshaw et al., 2022). Many recent tetrapod species (snakes, and some lizards and amphibians) have secondarily lost the functional middle ear system, leaving the mechanism of their airborne hearing unclear, as is also the case in the early tetrapods (see review in Capshaw et al., 2022). For example, in anuran amphibians, 38 instances of secondary loss of middle ear functionality (in the so-called ‘earless’ frogs; Jaslow et al., 1988) have been described (Pereyra et al., 2016), many of these in the bufonid family. ‘Earless’ frogs usually have an inner ear that is comparable to that of ‘eared’ species (but see a counter-example in Goutte et al., 2017). It has been known for some time that anurans generally were sensitive to low-frequency sound (below 400 Hz), although their eardrum shows very little response to these sounds (Wilczynski et al., 1987; Jørgensen and Christensen-Dalsgaard, 1997a,b; Lombard and Straughan, 1974; reviewed by Christensen-Dalsgaard, 2005). Also, several studies have shown that anuran low-frequency auditory fibers are very sensitive to substrate vibrations (Christensen-Dalsgaard and Jørgensen, 1996; Christensen-Dalsgaard and Narins, 1993; Jørgensen and Christensen-Dalsgaard, 1991; Yu et al., 1991). A comparison of the sensitivity of earless toads with similar-sized ‘eared’, or tympanate, species showed comparable sensitivity below 900 Hz (Womack et al., 2017; see below). Studies of airborne hearing in atympanate snakes (Christensen et al., 2012) and salamanders (Capshaw et al., 2020) have shown similar sensitivities to low-frequency sounds (reviewed in Capshaw et al., 2022, 2023).

Thus, in animals that lack tympanic middle ears to transform airborne sound into mechanical vibrations in the inner ear fluids, the inner ear may be stimulated by sound-generated translation of the head or body. This bone conduction mechanism is phylogenetically widespread, observed in lungfish, salamanders and snakes, and therefore may be a common pathway for sound detection in the absence of a functional middle ear.

In this study, we first evaluated an analytic model for translation of a cylindrical object by sound, based on Morse (1986). This model is an approximation that only works at low frequencies and predicts a vibration velocity that only depends on density. We next compared this model with a finite-element model of the same system, which allowed us a systematic evaluation of the dependence of vibration velocity on frequency and size. Finally, we compared the analytical model with empirical findings in both tympanate and atympanate vertebrate species. We propose that, in animals for which the size of the receiver (the head) is small compared with the wavelength of sound, translation by sound may be a general mode for transferring airborne acoustic energy to the inner ear.

**Table:** 

**List of symbols and abbreviations**	
*A*	acceleration
*a*	radius
*c*	sound velocity
*f*	frequency
*k*	wave number
*p*	sound pressure
*v*	vibration velocity
*v*_p_	particle velocity
*Z*	specific impedance
λ	wavelength
ρ	density

## SOUND AS A MECHANICAL FORCE

### Underwater sound

Underwater sounds relevant to most aquatic non-mammalian vertebrates tend to be below 5 kHz, so the wavelength is usually much larger than the animal. The acoustical size, defined as *ka*, the product of wavenumber *k* (=2π/λ) and radius *a* of the receiver (i.e. proportional to the ratio of radius to wavelength), is therefore less than 1. Because the density of the animal is roughly equal to the density of the surrounding water, the animal has nearly neutral buoyancy. The effect of acoustic stimulation at low *ka* is that the animal will be pushed and pulled by the sound pressure, generating a translational movement in the animal with the same particle velocity as the surrounding medium. In fish, the inner ear sensory cells are stimulated by inertial movement of the comparatively dense otoliths relative to this body motion. The particle velocity amplitude for a 1 Pa sound is *v*_p_=*p*/ρ*c*=0.6 μm s^−1^, where *p* is sound pressure, ρ is density and *c* is sound velocity. This particle velocity at 1 Pa corresponds to displacements below 1 nm for frequencies above 105 Hz. Displacements in this range are close to hair cell thresholds and suggest that sound sensitivity based on ‘unaided’ particle motion detection in water will be limited to short ranges, as sound pressure decreases with distance by the inverse square law.

### Airborne sound

#### An analytical model

The physical interaction between airborne sound and the body is different for a terrestrial animal because of the impedance difference between its tissue and the surrounding medium. Thus, a large fraction of sound energy will be reflected by the body of the animal, even when the animal is small compared with the wavelength of the incident sound (i.e. *ka*<1). Additionally, unlike aquatic organisms, terrestrial organisms are not suspended in the surrounding medium with neutral buoyancy and so friction against the ground will limit the induced movement. Morse (1986) developed an analytical model for the sound-induced (friction-less) whole-body movement of a small cylindrical object in air (see Appendix). When *ka*<<1, the model predicts that the induced velocity parallel and antiparallel to sound direction is largely independent of frequency (*f*) and can be approximated by *v*=2*p*/ρ*c* and acceleration *A*=(4π*fp*)/ρ*c* (Morse, 1986, correcting for a 2π error in the derivation; see Appendix), resulting in a vibration velocity of *v*=5.5 μm s^−1^ Pa^−1^ for an object density of 1060 kg m^−3^ (density of muscle tissue) and sound velocity 343 m s^−1^. Thus, the induced vibrations would be approximately 9 times larger in air than in water at the same sound pressures in the two media.

Sensitive eardrums, for example in mammals (Christensen-Dalsgaard and Manley, 2013), vibrate at approximately 1 mm s^−1^ Pa^−1^ in air, so if the vibration velocity in an atympanate animal was conveyed effectively to the inner ear, the sensitivity would be reduced approximately by a factor of 200 (46 dB) compared with the sensitivity of tympanate animals. The velocity declines with increasing *ka*, and the approximation is not valid for *ka*>1. Note that the model does not include effects of friction and mechanical parameters (e.g. elasticity, etc.) of the object.

#### Finite-element model

**Fig. 1.**
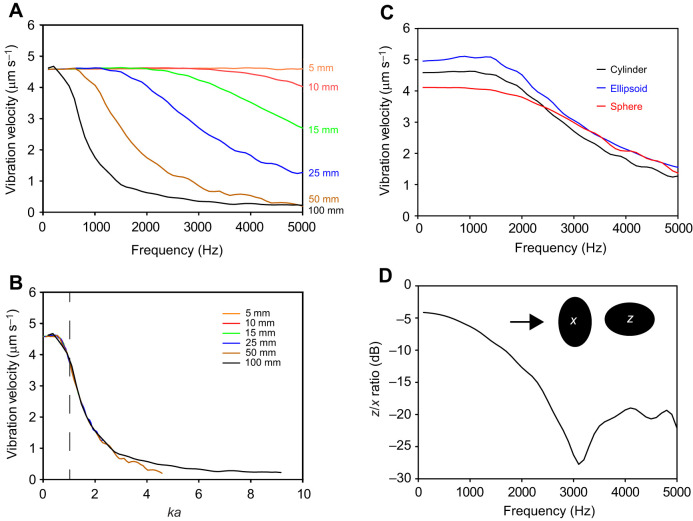
**Finite-element model of translation by sound.** (A,B) Finite-element model of the induced motion of a cylinder of different sizes translated in the direction of sound propagation in a sound field (1 Pa), in B shown as a function of *ka* (the dashed vertical line is *ka*=1). (C) Model of sound-induced motion of a cylinder, ellipsoid and sphere with the same radius (25 mm). (D) Ratio of vibration in ellipsoid with sound direction (arrow) perpendicular to the longitudinal axis (*x*) or parallel to the long axis (*z*).

Because the model is an approximation that only holds for low *ka*, we have tested its predictions by comparison with a simple finite-element (FE) model generated using COMSOL Multiphysics software (see details of the model in Supplementary Materials and Methods). The model object is a small cylinder with the same density as muscle (1060 kg m^−3^), ensonified by a plane sound wave with a sound pressure of 1 Pa. We modeled the responses of different sized cylinders with similar aspect ratios (1.5:1 length:diameter), spheres and ellipsoids, ranging from the head sizes of very small frogs (head diameter 0.01 m, length 0.015 m) to larger animals (head diameter 0.2 m, length 0.3 m). Fig. 1A shows the result of these model responses for different sizes of cylinders. The induced vibration velocities are maximal in the direction of wave propagation and have a constant amplitude of approximately 5 μm s^−1^ at low frequencies and a 3 dB cutoff frequency that ranges from above 5000 Hz for the smallest cylinders (i.e. comparable to small frogs or salamanders) to approximately 650 Hz for a radius of 100 mm, corresponding to the head size of a large tetrapod. Thus, the 3 dB decline depends on the size of the object, and when the response is plotted as a function of *ka*, the curves for different object sizes are almost identical (Fig. 1B). Therefore, the vibrational response of the cylinder to an impinging sound pressure wave depends only on *ka*, which could be understood as the acoustical size of the object, i.e. the relation of size to wavelength (Capshaw et al., 2022). At a *ka* of 1, velocity declines by 1.6 dB compared with the maximal amplitude, and a 3 dB decline is found at approximately *ka*=1.2. This decline increases with *ka* as the fraction of reflected sound energy increases.

The translation response also depends to a lesser extent on object shape. This was also tested by FE models by comparing the translation response of cylinders, spheres and ellipsoids with similar radii of 0.025 m. The result is shown in Fig. 1C. The three shapes show a similar decline with frequency, but the low-frequency plateau is lower for the sphere model than for the two other models. However, all fall in the range of 4–5 μm s^−1^ Pa^−1^.

Finally, the translation response is direction-dependent (Fig. 1D). The ellipsoid model (radius 25 mm) was stimulated across frequencies parallel (*z*) and perpendicular (*x*) to the major axis. The resulting vibration amplitude was smaller when sound direction (arrow) was perpendicular to the major axis (*x*) of the ellipsoid, as shown by the ratio of vibrations in the two conditions.

These FE models allow us to predict that this mode of acoustic stimulation should be sufficient to generate a physiological response in the inner ear of a small-bodied animal in air. If we analogize an animal's head to a simple cylinder, aerial sound pressures at frequencies below 3 kHz will be sufficient to generate comparable vibration velocities among animals with head widths smaller than 15 mm. This response drops off rapidly for larger head sizes (25 mm and larger), such that only very low frequencies (0.01–1 kHz) are capable of inducing body vibrations in larger animals. We additionally predict that this mode of sound energy transduction will be most efficient for sound waves that propagate perpendicular to the longitudinal plane of the animal's head, or along the inter-aural axis (Fig. 1D). These predictions will be tested by the experimental data that follows from four groups of atympanate tetrapods: salamanders, earless frogs, snakes and lungfish. In all cases, sound and vibration sensitivity (to whole-body vibration) has been measured and can be compared with model predictions.

## EXPERIMENTAL DATA

The analytical model, as well as the FE model, predict a simple relationship between sound level and induced body vibrations, with a baseline vibration response of approximately 5 μm s^−1^ Pa^−1^ at low *ka*. Thus, model predictions can be compared with experimental data, where sound thresholds have been measured in combination with measurements of either vibrations of the animal's head or whole-body vibration thresholds. The fundamental hypothesis is that if sensitivity to sound pressure can be explained by sensitivity to sound-induced vibrations, sound thresholds recalculated as head vibration thresholds would be comparable to vibration thresholds. Conversely, if the calculated head vibration thresholds are considerably lower than the whole-body vibration thresholds measured experimentally, it would suggest higher sound sensitivity than explained by the translation model, as it would be the case, for example, in an animal with a functioning tympanic ear. Here, the calculated vibration thresholds would be more than 40 dB lower than the vibration thresholds.

Using the analytical model of 5 μm s^−1^ Pa^−1^ for sound-induced translation at low *ka*, vibration velocities (in dB re. 1 mm s^−1^) at the sound thresholds are calculated simply by subtracting 140 dB from the sound thresholds (in dB re. 20 μPa). In the studies reported below, sound and vibration thresholds were measured either by auditory evoked responses or in one case (eared frogs) from single-unit responses.

### Salamanders

Salamanders have highly reduced middle ears that lack the tympanum, middle ear cavity and Eustachian tubes, which, in frogs, connect the two ears through the mouth cavity. Acoustic energy enters the oval window of the salamander inner ear via two middle ear elements, the ossified stapes (columella) and the cartilaginous operculum, which articulates with the stapes and has a muscular connection to the pectoral girdle (Kingsbury and Reed, 1908; Monath, 1965; Smith, 1968). Capshaw et al. (2020) measured sound and vibration thresholds using auditory brainstem responses in eight species – two ambystomatid species: *Ambystoma opacum* and *A. tigrinum*, and six plethodontid (lungless) species: *Desmognathus fuscus* sp., *Eurycea cirrigera*, *E. lucifuga*, *Gyrinophilus porphyriticus*, *Plethodon cinereus* and *P. glutinosus*. Additionally, sound-induced vibrations of the heads of salamanders were measured using laser vibrometry. Threshold sensitivities to airborne sound ranged from 57 to 85 dB SPL, and the most sensitive frequencies ranged from 100 to 250 Hz. For each species, thresholds to vibrations were almost uniform (in terms of acceleration) up to approximately 300 Hz, but varied across species from −55 to −35 dB re. 1 m s^−2^.

**Fig. 2.**
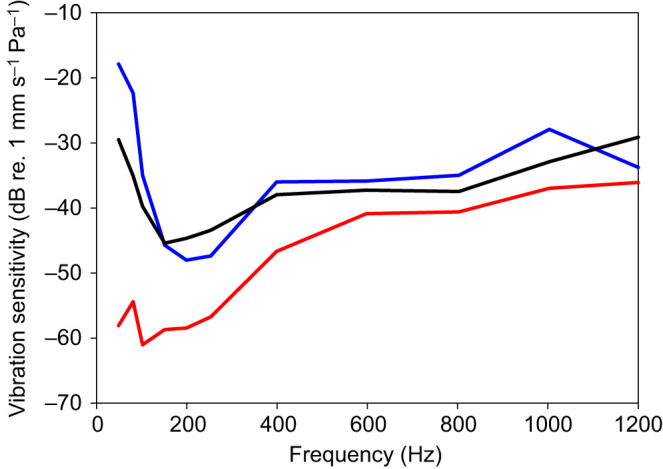
**Averaged data from seven salamander species showing thresholds to sound-induced head vibrations measured at the sound thresholds with laser vibrometry (blue curve), compared with seismic vibration thresholds (black curve) and analytical model prediction of induced head vibrations based on the sound thresholds (red curve).** Data from Capshaw et al. (2020).

Using laser Doppler vibrometry data, sound thresholds may be referred to sound-induced head vibration and recalculated as vibration thresholds (Fig. 2). Because all species were relatively small (largest head radius 1 cm), and their auditory sensitivity was largely confined to frequencies below 400 Hz, we estimated that the *ka* for salamanders is <0.07 and we show averaged data from all species in Fig. 2. The measured head vibrations (blue curve) at sound thresholds are comparable to the vibration thresholds (black curve). The analytical model head vibrations predicted from the sound thresholds (red curve) are approximately 10 dB lower than the measured head vibrations.

A partial explanation of this discrepancy is that the model does not include friction between the animal and the ground, which must limit the movement of the head. An additional source of friction could be between the head and the rest of the body, but because both the head and the body were in contact with the ground, this source may be of minor importance. Despite the observed discrepancy, this model can be used to estimate the amplitude of whole-body vibrations induced in salamanders by airborne sound. Comparisons of model data to sensitivity thresholds can then be used to approximate the efficacy of sound-induced vibrations to evoke a response in the auditory system. In summary, sensitivity to both sound and vibrations may be attributed to sound-induced head vibration in salamanders, and the effective stimulus reaching the inner ear can be estimated as −10 dB re. 5 μm s^−1^ Pa^−1^, or 1.6 μm s^−1^ Pa^−1^.

### Earless frogs

**Fig. 3.**
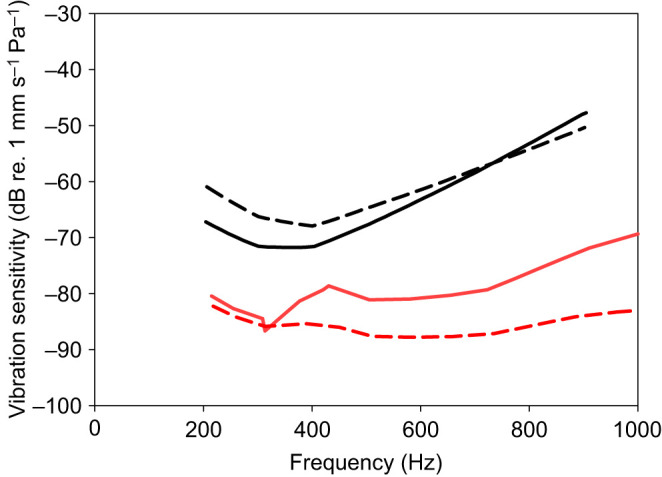
**Average vibration thresholds (to dorso-ventral vibration) of earless (black line, data from 4 species) and eared (black dashed line, data from 6 species) toads.** The model curves show vibration sensitivity predicted from the thresholds to airborne sound, assuming the sound translation model described in the text (red line: earless, red dashed line: eared). From Womack et al. (2017), redrawn.

Earless frogs are highly diverse in the degree of reduction to their middle ears; at minimum, an earless species lacks tympana, but may also lack the tympanic annulus and stapes (Pereyra et al., 2016). Despite lacking some or all components of the tympanic middle ear, many earless frogs vocalize and presumably detect these acoustic signals via extratympanic pathways (Boistel et al., 2011; Lindquist et al., 1998; Womack et al., 2018), although some species may have lost sensitivity to the acoustical signals and may use visual cues generated by the vocalizing male (Goutte et al., 2017). In a study of ten eared and earless bufonid species, Womack et al. (2017) presented data on sensitivity to sound and vibration stimuli. Sound thresholds recorded in eared toads are up to 20 dB more sensitive than the earless toads for frequencies above 900 Hz; however, vibration sensitivity (Fig. 3) and low-frequency (<300 Hz) sound sensitivity are similar among eared (*Rhinella tacana*, *R. alata*, *R. leptoscelis*, *R. spinulosa*, *R. horribilis* and *Rhaebo haematiticus*) and earless (*R. arborescandens*, *R. festae*, *R. yunga* and *Osornophryne guacamayo*) toads studied. Above approximately 300 Hz, eared toads are slightly more sensitive to sound than the earless, probably reflecting increased stimulation via the tympanic pathway at higher frequencies, and the difference in sensitivity increases with frequency. In contrast, earless frogs have lower thresholds to vibrations at frequencies below 700 Hz. Womack et al. (2017) did not measure sound-induced vibrations in their animals, but analytical model calculations of the induced vibrations at the sound thresholds (red curves in Fig. 3) result in a prediction of sound-induced vibration thresholds that are around 20 dB lower than the measured vibration thresholds (black curves in Fig. 3). This may be caused by the direction of stimulation (dorso-ventral for vibration stimulation, lateral incidence for sound), but also by frictional losses not accounted for by the model. The difference between eared and earless frogs is due to increased sensitivity of the tympanic ear at higher frequencies.

### Eared frogs

Eared, terrestrial frog species have well-developed tympanic middle ears with flexible tympana coupled to the inner ear via the extracolumella and columella (stapes), in addition to the operculum, which articulates with the stapes in the oval window (Mason et al., 2015). Additionally, the middle ear cavities of the two ears are connected to the mouth cavity via the Eustachian tubes. Therefore, the inner ear of tympanate frogs may be stimulated by many different pathways for acoustic energy, including directly through the tympanum or the opercularis system, or indirectly through the lung–ear pathway (Ehret et al., 1990, 1994; Lee et al., 2021; Mason, 2007; Narins et al., 1988). These non-tympanic pathways are shown to be most effective at low frequencies (Wilczynski et al., 1987; reviewed in Christensen-Dalsgaard, 2005). Also, comparison of sound and vibration sensitivity of single auditory fibers in the eared frog *Rana temporaria* showed that all low-frequency fibers had a dual sensitivity to sound and dorso-ventral vibrations (Christensen-Dalsgaard and Jørgensen, 1996).

**Fig. 4.**
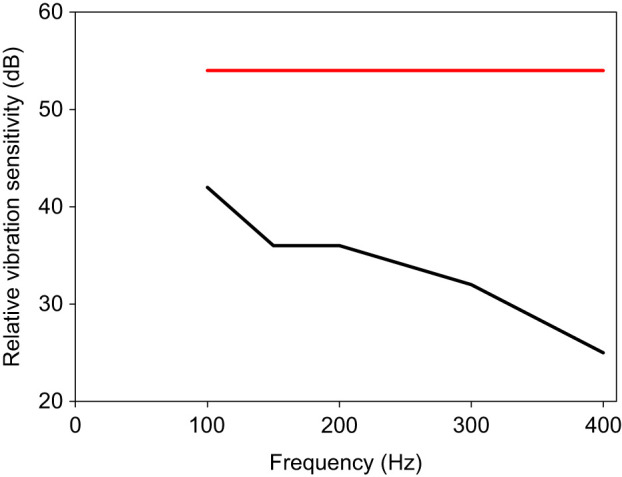
**Average relative vibration sensitivity in single auditory nerve fibers in the grass frog (*Rana temporaria*).** Relative vibration sensitivity is the ratio between particle velocity of sound and induced particle velocity. Because the analytic model predicts 5 μm s^−1^ Pa^−1^, and the sound particle velocity in air is approximately 2.5 mm s^−1^ Pa^−1^, the ratio is 500 or 54 dB (the red curve). The black curve shows the ratio between sound particle velocity thresholds and thresholds to dorso-ventral vibrations in experimental data from 106 fibers. Data from Christensen-Dalsgaard and Jørgensen (1996).

The average relative vibration sensitivity, measured as the sensitivity difference between vibration stimulation, vibration velocities and sound stimulation particle velocities (Fig. 4), varied systematically from 42 dB at 100 Hz (i.e. sound particle velocities were 42 dB less efficient than dorso-ventral vibration velocities) to 25 dB at 400 Hz, most likely showing increased sensitivity for tympanic vibrations with increased frequency (Christensen-Dalsgaard and Jørgensen, 1996). If the frog was stimulated solely by induced vibrations, the analytical model predicts a frequency-independent relative vibration sensitivity of 54 dB (the ratio between sound particle velocity – 2.5 mm s^−1^ Pa^−1^ – and induced velocity of 5 μm s^−1^ Pa^−1^). The relative vibration sensitivity at 100 Hz is 12 dB lower, suggesting that also in this case the model predicts higher vibration amplitude than was actually observed. The difference is most likely caused by friction, but alternatively, the sound stimulation may be more efficient than vibration stimulation even at low frequencies (suggesting tympanic input even at low frequencies). Additionally, the direction of stimulation is different for sound and vibration stimulation (dorso-ventral for vibrations, lateral for sound particle velocities).

### Snakes

**Fig. 5.**
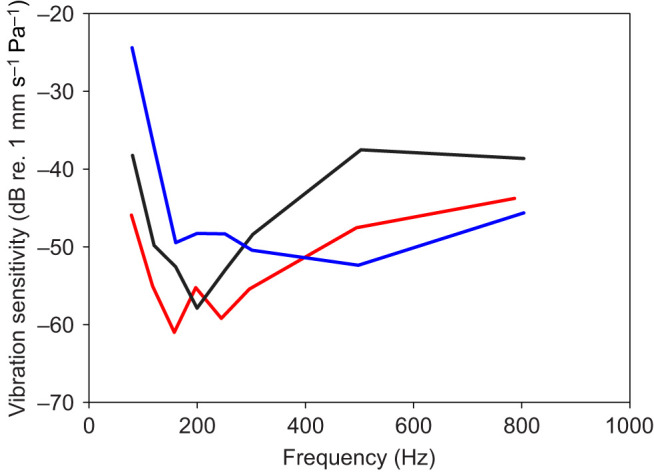
**Average sound and vibration thresholds of royal pythons, *Python regius*.** Sound thresholds are referred to the measured sound-induced head vibrations (blue). The red curve shows the model-predicted induced vibrations, and the black curve is experimentally measured vibration thresholds. Redrawn from Christensen et al. (2012).

The middle ear of snakes is modified compared with other terrestrial vertebrates as the columella/stapes is connected via the quadrate to the lower jaw, and the other tympanic structures have been lost. This is probably an adaptation to detect seismic vibrations. Christensen et al. (2012) studied auditory brainstem responses in the royal python, *Python regius*, to both sound and vibration stimuli and measured the sound-induced head vibrations (Fig. 5). Sensitivities to sound and vibration are most similar from 100 to 300 Hz. The lowest threshold for sound was 78 dB SPL measured at 160 Hz, corresponding to a model velocity of −62 dB re. 1 mm s^−1^. At 500 Hz, the sound threshold level increases to 92 dB SPL, corresponding to a model acceleration of −48 dB re. 1 mm s^−1^. Interestingly, analytical thresholds predicted by the model for frequencies above 400 Hz are higher than the sound-induced head vibration thresholds, suggesting a more efficient stimulus pathway for sound at these frequencies. Although non-tympanic, snakes have a movable columella (Christensen et al., 2012) that might cause the increased sound sensitivity at higher frequencies, but generally, sound-induced head vibration drives dual responses to sound and vibration.

### Lungfish

**Fig. 6.**
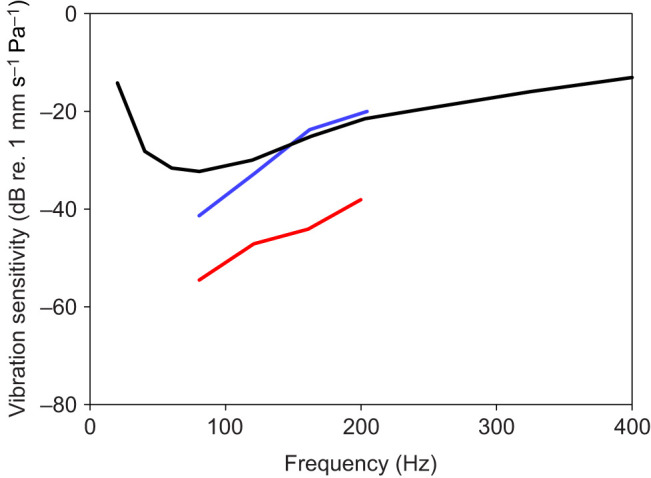
**Thresholds to airborne sound and vibration in African lungfish, *Protopterus annectens*.** The sound thresholds (blue) refer to the sound-induced head vibrations for comparison with the vibration thresholds (black). The model head vibrations calculated from the sound thresholds are shown in red. Redrawn from Christensen et al. (2015).

Lungfish hearing is mediated by the otolithic organs of the inner ear that detect underwater sound as particle motion and pressure (Christensen-Dalsgaard et al., 2011; Christensen et al., 2015). The lungfish ear is probably similar to the ear of the early tetrapods (Clack, 2002), and therefore its hearing in air is particularly interesting. Lungfish hearing in air is rather insensitive and restricted to low frequencies, most likely because it is limited by the frequency response of the otolithic hearing organs. Christensen et al. (2015) measured hearing sensitivity of the African lungfish (*Protopterus annectens*) in air using auditory brainstem responses (Fig. 6). The lowest detection threshold for airborne sound was 86 dB re. 20 μPa, measured at 80 Hz, and the threshold increased to approximately 100 dB re. 20 μPa at 200 Hz. For lungfish, the head radius is approximately 0.05 m, so *ka*<1 for frequencies below approximately 1 kHz. The analytical model calculation predicts a vibration amplitude of −54 dB re. 1 mm s^−1^ at 80 Hz and −40 dB at 200 Hz (Fig. 6, red line). Again, the model predicts thresholds that are lower than what is actually measured, because the predicted head vibrations are greater than the measured vibrations.

### Directional hearing

**Fig. 7.**
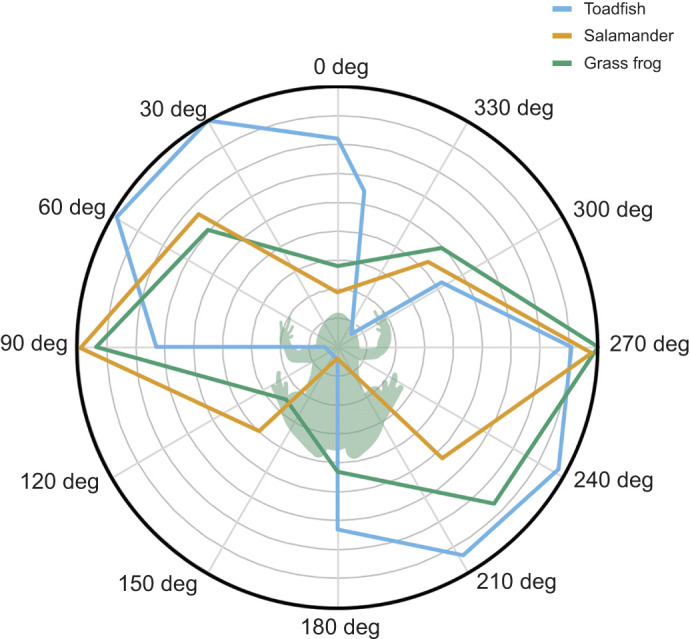
**Low-frequency auditory directionality of three vertebrates stimulated in the horizontal plane.** Polar plots are normalized to allow for comparisons among different methods; 0 deg indicates the rostral direction for all taxa represented. Similar patterns of directional sensitivity may be observed across taxa as a result of the inherently directional response pattern of oppositely polarized hair cells. Blue: directional response patterns for a primary saccular afferent in a toadfish, *Opsanus tau*, from 100 Hz stimulation (Edds-Walton and Fay, 2005). Note that toadfish saccular afferents show sensitivity to many other sound source directions, depending on the innervated hair cell orientation. Orange: directionality of the auditory brainstem response of a salamander, *Eurycea lucifuga*, to a 200 Hz tone. Directional auditory sensitivity is represented by masking efficiency of a tone broadcast from different locations around the salamander, normalized to the ipsilateral response (Capshaw et al., 2021). Green: directional response of a low-frequency auditory nerve fiber in a grass frog, *Rana temporaria*, stimulated at 200 Hz (Jørgensen and Christensen-Dalsgaard, 1997a). Reprinted from Capshaw et al. (2022).

Measurement of directional hearing sensitivity in salamanders using auditory evoked responses (Capshaw et al., 2021) and in grass frogs using single-unit responses (Jørgensen and Christensen-Dalsgaard, 1997a,b) showed a similar, figure-eight response at low frequencies, with the strongest responses from lateral stimulus directions. A comparison of the directionality of single-unit fibers in salamanders, frogs and toadfish is shown in Fig. 7 (from Capshaw et al., 2022; note that the toadfish was stimulated by directional vibration and the frogs and salamanders by sound). All three species show directional responses, and the directionality to sound in the frog and salamander may be caused by sound-induced directional vibration, equivalent to the directional stimuli of the toadfish. These responses were thought to be caused by a directional component in fluid movement in the otic capsule, so lateral stimulus directions produced larger fluid movements. However, the model studies reported here (Fig. 1D) suggest that sound translation of the body in itself may be directional. The two explanations are not mutually exclusive, and the combination may result in improved directionality in the afferent neurons.

## DISCUSSION

The experimental data reviewed here show similar ranges of sound sensitivities among all atympanate animals studied. Auditory sensitivity is restricted to low frequencies (defined by *ka*, the acoustical size of the animal, with *ka*<1) and can in most cases be explained by sensitivity to sound-induced vibrations of the head of the animal. We used an analytical model to predict the ability of sound to cause translational movement in these atympanate animals, approximated as a simplified cylinder with the density of muscle tissue, and then compared the results with an FE model of the cylinder. The two models predicted similar amplitude of cylinder movement. In addition, the FE model showed that the movement amplitude solely depended on *ka* and sound pressure (Fig. 1B) for same-shape elements, and that the sound-induced movement is directional (Fig. 1D).

Compared with experimental data (Figs 2–6), in all cases the analytical and FE models predicted 10–15 dB lower sound thresholds than actually measured in all experiments. The same difference is found in comparison with thresholds based on measurements of head vibrations by laser vibrometry (Figs 2 and 6). Thus, although this simple bone conduction mechanism for sound detection can generate sufficient amplitude vibrations in atympanate animals to explain the auditory thresholds, the induced vibrations that reaches the inner ear are at least 10 dB lower than modeled, closer to 1 μm s^−1^ Pa^−1^. This value is probably a reasonable baseline estimate of sound and vibration stimulation equivalence and would result in a 60 dB advantage of a sensitive tympanic ear (1 mm s^−1^ Pa^−1^ vibration) in comparison with non-tympanic tetrapods.

The most likely explanation for the discrepancy between modeled and actual vibrations is that the model does not account for friction, which must be important, perhaps especially at high frequencies. Note that in the experiments reported here, the animals were anesthetized and lying flat with the head and body in contact with the substrate. However, under natural conditions (head raised), the motion of the head and body is likely not uniform, and the head will move relative to the body of the animal. Here, the important part of friction will be friction in the neck muscles and spine. Interestingly, the amphibians connect the operculum, a movable piece of cartilage next to the oval window, to the scapula and may exploit the relative movement of the head and body, as hypothesized by Capshaw et al. (2022).

Further, cranial vibrations may also contribute to non-tympanic hearing, as is the case in humans (Stenfelt, 2013), where structural vibrations dominate bone-conduction sensitivity above approximately 400 Hz. However, such vibrations will be strongly dependent on the anatomy and material properties of the head and might contribute at lower *ka* in other animals. Resonances in air cavities could also contribute to the vibrations of the head, as suggested by Boistel et al. (2011). Also, some energy may be lost in the transmission of vibration to the inner ear fluids (see below).

Our findings provide support for a general mechanism for sound reception without any specialized middle ear apparatus via translation of the head by sound. This mechanism would have been functional in the earliest tetrapods that may have had an ear configuration similar to that of the lungfish (Fig. 6). Interestingly, this mechanism for sound-induced head translation is also proposed for human bone conduction frequencies below 400 Hz (i.e. *ka*<0.62 assuming a head radius of 0.085 m) (Stenfelt, 2013), and a sound-induced translation of the human head of 3 μm s^−1^ Pa^−1^, as proposed by von Bekesý (1960), fit the measured human bone conduction thresholds at low frequencies reasonably well (see Sørensen et al., 2022).

Additionally, comparisons of sensitivity of eared and earless bufonids by Womack et al. (2017) (Fig. 3) show that, for low-frequency hearing, earless species are not really at a disadvantage relative to tympanate species, indicated by nearly equivalent thresholds to sounds below 900 Hz. Thus, this bone conduction mechanism for hearing may explain the relaxed selection pressures for functioning middle ears in the anurans, which has allowed loss of a functional middle ear in many species (Pereyra et al., 2016). In anuran amphibians, the structures of the tympanic middle ear form relatively late during development, and reduction or loss of the tympanic middle ear may be correlated to developmental heterochronies associated with pedomorphosis and/or miniaturization of body size and particularly common in amphibians (Hetherington, 1992; Pereyra et al., 2016, Womack et al., 2019). Especially in connection with miniaturization, an allometric downsizing of the tympanum would increase its stiffness and therefore reduce sensitivity to airborne sound and further relax the selection pressure for functional middle ears, trading part of the auditory sensitivity for early development (Capshaw et al., 2022).

The question still remains how the whole-body vibration stimulates the inner ear. Some of the mechanisms reported for human bone conduction, such as inertial movement of the middle ear ossicles that presumes movable middle ear ossicle(s), or high-frequency vibrations traveling through the cranial bone to the inner ear fluids, are not likely to function for low-frequency translation by sound. In fish, sound reception is facilitated by the inertial vibrations of the otoliths, but the otoliths of tetrapods, at least in amphibians, are only sensitive in a restricted frequency range (Christensen-Dalsgaard and Jørgensen, 1988). Alternatively, inertia of the inner ear fluids has been proposed as the dominant mechanism for human bone conduction (Stenfelt, 2015). Sensitivity to fluid inertia depends on the size and orientation of pressure release windows in the inner ear and is a very general mechanism that would function in a variety of tetrapods, for example in amphibians, where whole-body vibration does stimulate the low-frequency hearing organ (Jørgensen and Christensen-Dalsgaard, 1991; Christensen-Dalsgaard and Narins, 1993; Christensen-Dalsgaard and Jørgensen, 1996; see also review in Capshaw et al., 2022).

### Conclusions

Sound-induced vibration of around 1 μm s^−1^ Pa^−1^ for tetrapods with low acoustical size, *ka*, can provide a baseline auditory sensitivity that explains terrestrial hearing abilities observed in diverse animals without specialized middle-ear structures. Also, small animals, which have eardrums that are less responsive at low frequencies, may use this baseline sensitivity for low-frequency hearing, perhaps even extending to infrasound sensitivity (Zeyl et al., 2020). The baseline sensitivity would be expected to be approximately 60 dB below that of a sensitive tympanic ear.

Before the origin of the tympanic middle ear, sound-induced vibrations likely were the mechanism for stimulation of the ear in early tetrapods and could potentially even provide directional information (Fig. 1D; see also Capshaw et al., 2022). This may have provided an important foundation for pressure sensitivity on land, until a series of modifications leading to a functional middle ear increased sensitivity to airborne sound (Christensen-Dalsgaard and Carr, 2007). These modifications are proposed in a three-step model of middle ear evolution by Christensen-Dalsgaard and Manley (2013): a reduction of otolith covering in the sensory macula, development of movable elements such as columella or operculum and, finally, formation of columella–tympanum connection.

## Supplementary Material

10.1242/jexbio.251719_sup1 Supplementary information

## APPENDIX

### Morse: force on cylinder

This calculates the acoustical force on an object – a small cylinder of unit length (Morse, 1986, p. 352). The approximations are for low *ka*, i.e. objects that are small compared with the wavelength of sound. It is usually stated that such objects are ‘transparent’ to sound, but locally, because the object motion must be equal to the particle velocity of the medium bordering the object, a part of the sound wave is reflected. Therefore, the equations below incorporate the (frequency-dependent) reflection coefficients.

The force is pressure times area:

(rmA1)

(rmA2)

where *a* is the radius and *c*_m_, γ_m_ are the reflection coefficient amplitudes and phases of the cylinder (Morse, 1986, 26.6, p. 301):

(rmA3)

At low *ka*,  coefficients are small when *m*>1, and a useful approximation is therefore to use only the two first elements (*c*_0_ and *c*_1_), i.e.:

(rmA4)

The first (*c*_0_) term in the integral is constant in φ, so the integral is zero (integrating a cos function over a whole cycle), resulting in the integral:

(rmA5)

(rmA6)

(rmA7)

The amplitude is .

Acceleration is  where *m* is mass, ρ is density and *V* is volume, *V*=π*a*^2^*l*.

Thus, , velocity  and displacement .

Note that the formula stated by von Békésy (1960, p. 171) for a sphere at low *ka* is , a factor of 2 lower.
