# Supplementary material for: A general mechanism of airborne hearing in recent and early non-tympanate tetrapods
Source: J Exp Biol. 2026 Jun 3;229(11):jeb251719. doi: 10.1242/jeb.251719 (PMC13286356; doi:10.1242/jeb.251719)

Supplementary Materials and Methods

Comsol-model.

The finite-element models of ensonified objects were made in COMSOL Multiphysics, v. 5.6 (COMSOL A/S, Lyngby, Denmark). We used simple geometric shapes – cylinders, spheres and ellipsoids, with varying radii and (for cylinders and ellipsoids) the same length/diameter ratio (1.5). The model simulated the shapes suspended in air (frictionless) and ensonified by plane sound waves from different directions (parallel and perpendicular to the long axis). The model was placed in the center of a spherical domain with radius 0.4 m and non-reflecting boundary.

The meshing was triangular, using a physics-controlled mesh with 'finer' resolution (Comsol default setting, changed to 'extra fine' setting for the three smallest objects). The quality of the meshing was controlled by the smoothness of the model output at the highest frequencies. For the smallest objects, the extra fine mesh was necessary to avoid ripples in the frequency response curve.

The material of the shapes had a density like muscle tissue (1060 kg/m^3), but to avoid internal vibrations in the structure that would obscure the effects of whole-body movement we made the material unrealistically stiff (Youngs modulus 45 * 10^9 Pa), more than 1000 times higher than normal tissue.

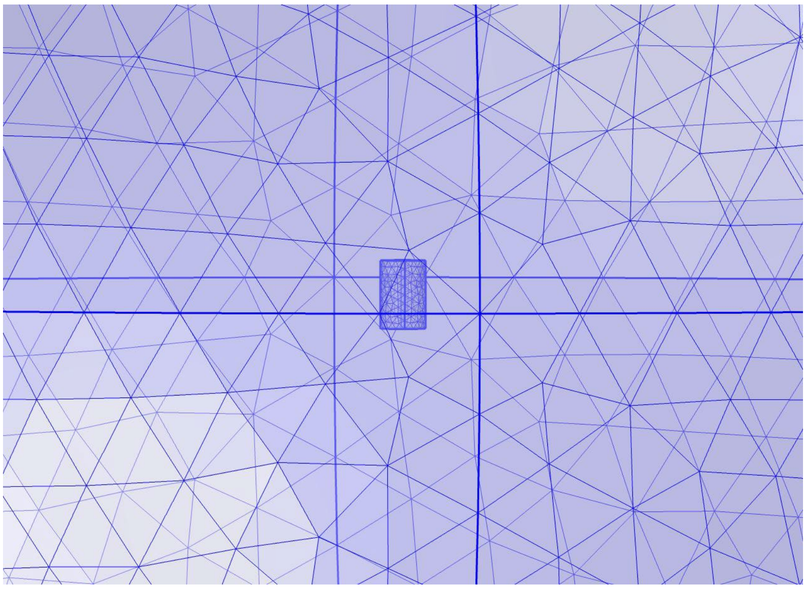

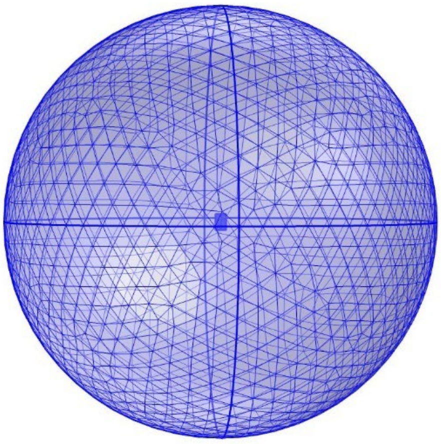

Supplement: Supplementary information [file jexbio-229-251719-s1.pdf]
